# Impaired Neurobehavioural Performance in Untreated Obstructive Sleep Apnea Patients Using a Novel Standardised Test Battery

**DOI:** 10.3389/fsurg.2018.00035

**Published:** 2018-05-18

**Authors:** Angela L. D'Rozario, Clarice J. Field, Camilla M. Hoyos, Sharon L. Naismith, George C. Dungan, Keith K. H. Wong, Ronald R. Grunstein, Delwyn J. Bartlett

**Affiliations:** ^1^CIRUS, Centre for Sleep and Chronobiology, Woolcock Institute of Medical Research, University of Sydney, Sydney, NSW, Australia; ^2^School of Psychology, Faculty of Science, Brain and Mind Centre and Charles Perkins Centre, University of Sydney, Sydney, NSW, Australia; ^3^Sydney Medical School, University of Sydney, Sydney, NSW, Australia; ^4^Department of Respiratory and Sleep Medicine, Royal Prince Alfred Hospital and Sydney Local Health District, Camperdown, NSW, Australia

**Keywords:** polysomnography, sleep-disordered breathing, inter-individual variability, cognitive impairment, vigilance, attention

## Abstract

**Objective/Background:**

Although polysomnography (PSG) is the gold-standard measure for assessing disease severity in obstructive sleep apnea (OSA), it has limited value in identifying individuals experiencing significant neurobehavioural dysfunction. This study used a brief and novel computerised test battery to examine neurobehavioural function in adults with and without OSA.

**Patients/Methods:**

204 patients with untreated OSA [age 49.3 (12.5) years; body mass index, [BMI] 33.6 (8.0) kg/m^2^; Epworth sleepiness scale 12 (4.9)/24; apnea hypopnea index 33.6 (25.8)/h] and 50 non-OSA participants [age 39.2 (14.0) years; BMI 25.8 (4.2) kg/m^2^, ESS 3.6 (2.3)/24]. All participants completed a computerised neurobehavioural battery during the daytime in the sleep clinic. The OSA group subsequently underwent an overnight PSG. The 30 min test battery assessed cognitive domains of visual spatial scanning and selective attention (Letter Cancellation Test), executive function (Stroop task) and working memory (2- and 3-Back tasks), and a validated sustained attention task (psychomotor vigilance task, PVT). Group differences in performance were compared. Associations between disease severity and performance were examined in the OSA group.

**Results:**

After controlling for age, gender and education, OSA patients demonstrated impaired performance on the Stroop-Text, 2 and 3-Back tasks, and the PVT compared with the non-OSA group. OSA patients had worse performance on the LCT with fewer average hits albeit with better accuracy. Some OSA polysomnographic disease severity measures were weakly correlated with performance.

**Conclusions:**

This brief test battery may provide a sensitive, standardised method of assessing daytime dysfunction in OSA.

## Highlights

- A brief computerised test battery assessed cognitive function in untreated OSA
- OSA patients demonstrated impaired neurobehavioural performance in all domains
- Polysomnographic disease severity measures were weakly correlated with performance
- The battery provides a sensitive method of assessing daytime dysfunction in OSA

## Introduction

Obstructive sleep apnea (OSA) is a highly prevalent sleep disorder affecting 25–50% of middle-aged adults in the general community (1). It is characterised by repeated episodes of upper airway obstruction, intermittent hypoxemia/hypercapnia and repeated arousals during sleep. Untreated OSA results in a substantial health care burden with varying levels of excessive daytime sleepiness and neurobehavioural dysfunction (2, 3), increased incidence of workplace accidents (4) and more than a 2-fold increased motor vehicle crash risk (5). Neuropsychological tests show deficits in the cognitive domains of vigilance and attention, visuospatial abilities, executive functions and some components of memory in OSA, while data for other domains remain equivocal (6). There is currently no standardised neuropsychological test battery for assessing daytime dysfunction which can be briefly administered in a sleep laboratory setting. Current tests assessing sleepiness such as the multiple sleep latency test (MSLT) or maintenance of wakefulness test (MWT) are time consuming and expensive. These specialised tests are not routinely indicated to evaluate OSA or treatment effectiveness unless associated with a safety issue (7).

Clinicians predominantly rely on both traditional PSG-derived measures to assess OSA disease severity and self-report questionnaires to evaluate daytime dysfunction. However disease severity measures are inconsistently (8) or weakly related to neurocognitive test performance (9). A recent meta-review provided evidence that sleep fragmentation measures were more strongly related to performance on tests of vigilance and attention than hypoxemia measures (6). Although hypoxemia may affect global cognitive functioning, no clear links were found between disease severity and performance assessing executive functions, memory, visuospatial capacity, language ability and psychomotor function (6). Continuous positive airway pressure (CPAP) therapy which is still the “gold-standard” treatment for OSA has limited or partial reversibility of cognitive deficits (10–12). These outcomes emphasise the need for early detection of sleep-disordered breathing and potential cognitive decline in OSA. Targeted treatment particularly in light of recent research identifying untreated sleep apnea as a significant risk factor for developing mild cognitive impairment or dementia is imperative (13).

Current neurobehavioural test batteries attempt to evaluate the impact of sleep breathing disorders on daytime functioning but differences between OSA and non-OSA groups are not clearly delineated (14–16). Differing methodologies, the timing and the type of neuropsychological tests administered, and characteristics of the patient groups studied e.g., age, daytime sleepiness levels and variable treatment histories; as well as different levels of CPAP compliance may account for the varied results in both case-controlled observational studies and in treatment interventions. Simple but sensitive tools which can be administered with ease in a clinical setting are needed to evaluate neurobehavioural dysfunction as well as treatment efficacy in OSA patients. While large (2.5–3 h in duration) test batteries provide comprehensive assessment with the potential for a greater understanding of the patterns of neurobehavioural deficits (17), shorter testing protocols may be more practical and cost-effective in the clinical setting and minimise patient fatigue. Easy access to neurocognitive batteries could potentially enable newly diagnosed patients reticent to undertake treatment to then become aware of their individual cognitive deficits. Detecting early changes in cognition may also be useful for the assessment of future risk of dementia in sleep apnea patients (18).

In this study, we compared the performance of treatment naïve OSA patients with a non-OSA group, using a short (30 min in duration) standardised computerised neurobehavioural test battery which assessed the salient cognitive domains known to be impaired in OSA: attention/vigilance, visuospatial abilities, executive functions and working memory. In a secondary analysis, we examined the association between PSG measures of disease severity and test battery performance.

## Materials and Methods

### Participants

This study is a secondary analyses using baseline performance data collected from OSA patients who participated in a large randomised trial comparing a psychoeducation intervention to improve CPAP adherence (Australia and New Zealand clinical trials registry number 12606000065594) (19). The Sydney South West Area Health Service Human Research Ethics Committee approved the protocol and volunteers provided written informed consent. Individuals were asked to participate in a study that would be valuable in helping them to use CPAP. Participants were recruited following a polysomnography-confirmed diagnosis of OSA (respiratory disturbance index ≥5/h. This index included apneas, hypopneas and respiratory-event related arousals) and sleep physician referral for a CPAP-titration study from three geographically distinct clinics in Sydney, New South Wales (Royal Prince Alfred Hospital, Royal North Shore Hospital and the Woolcock Institute of Medical Research). Recruitment took place between November 2007 and August 2009. Sleep physicians referred patients to the study on the basis of their diagnostic sleep study and the need to improve their current health and sleep. Patient data from *n* = 204 who met the inclusion criteria of a respiratory disturbance index ≥5/h confirmed by diagnostic PSG and who underwent neurobehavioural testing were included in the present analyses. Exclusion criteria for the OSA patients included any previous or current use of CPAP and non-fluency in both written and spoken English, or use of psychotropic medications. As part of the large randomised trial, participants were screened for any significant co-morbidity by the sleep physician prior to entry into the study. The physicians used their own judgement and considered any significant health conditions e.g., dementia, major neurological problems (e.g., stroke, epilepsy); severe mental health disorders (e.g., schizophrenia, major depression, bipolar disorder) prior to confirming eligibility to participate in the trial. During the eligibility screening visit, a family history of cardiovascular disease was taken and the presence of hypertension was recorded but hypertension was not an exclusion criteria.

For the non-OSA group, fifty healthy individuals without OSA were recruited from January to December 2009 specifically for this current study by general community advertisement and local newspaper. The University of Sydney Human Research Ethics Committee approved the protocol and all participants provided written informed consent. They were at low-risk of OSA [Multivariable Apnea Prediction Index (MAP index) value <0.5] (20). Exclusion criteria for the non-OSA group were: significant sleepiness (Epworth Sleepiness Scale score (ESS) >10) (21); clinical insomnia (Insomnia Severity Index [ISI] score >15) (22); history of sleep disorders, neurological disorders, major psychiatric disorders, other significant concomitant medical co-morbidities, or head injury; usage of medications affecting sleep or cognitive function; and colour-blindness.

The educational status of each participant was categorised as either: primary, secondary or tertiary level.

### Neurobehavioural Test Battery

**Figure 1:**
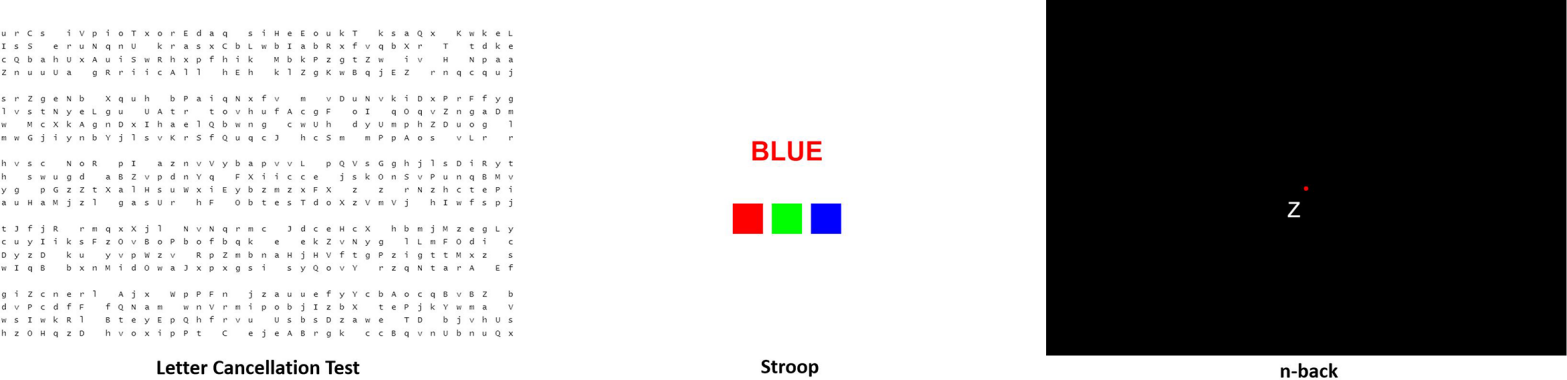
Screen shots of the computerised neurobehavioural tasks included in the test battery. Appropriate permissions for use of screen shots granted.

The test battery comprised two parts: (1) computerised neurobehavioural tasks modelled on conventional neuropsychological tests (23); and, (2) the hand-held 10 min psychomotor vigilance task (PVT) (24). The neurobehavioural tasks selected were based on cognitive domains which previously identified impairment in OSA: attention/vigilance, visuospatial abilities, executive functions and working memory. The computerised battery was developed on a web-based platform, and delivered on a conventional desktop personal computer with a 17-inch colour display, keyboard and mouse. Administration of the full test battery lasted approximately 30 min including the 10 min PVT. All participants first performed a practice session of the test battery in the presence of a researcher. Individual tasks were repeated until the participant felt comfortable with each task. The test battery was undertaken in a private testing room in the sleep clinic. The OSA patients stayed in the sleep clinic and had an in-lab overnight sleep study on the same evening following the performance testing which occurred in the afternoon. The non-OSA group did not undergo PSG. Itemisation of the tests incorporated within the neurobehavioural battery is detailed below (see Figure 1).

#### Letter Cancellation Test (LCT)

The LCT predominantly evaluates attention, concentration and visuospatial scanning ability or visuospatial neglect, as well as measuring accuracy of selective attention (23). A large field of letters is displayed on the computer screen, including a target CAPITAL LETTERS, d o u b l e s p a c e s, or a mixture of both (25). Participants scanned this field and were asked to mark as many capital letters displayed on the computer screen in 60 s using the mouse (this was performed twice – trials 1 and 2); then mark as many double spaces (trials 3 and 4), then as many capital letters and double spaces (trials 5 and 6). On the final screen (trial 7) a combination of the two targets were displayed and participants were asked to complete the test in their own time. Variables evaluated were the mean number (average performance on trials 1–6) of: (a) correctly marked targets (“average hits”); (b) missed targets (“average omissions”); and, (c) non-targets incorrectly marked (“average commissions”). Performance variables assessed during the final trial were final hits, finals omissions, final commissions and final trial duration.

#### Stroop Text and Stroop Colour

The Stroop test assesses the inhibition of dominant responses, and reflects the “higher-order” executive functions (23). It is a two-part test (Stroop-Text and Stroop-Colour) assessing reaction time to colours and words displayed and gauges cognitive interference which is impacted by the presentation of simultaneous conflicting information. Words (red, green, or blue) and three different coloured squares (red, green, or blue) were displayed on the computer screen. Participants were required to click on the coloured square that matched either the MEANING (Stroop-Text) or the COLOUR (Stroop-Colour) of the word presented. Each part of the test was 45 s in duration and involved multiple trials. Variables evaluated were percentage of correct total responses; and average response latency.

#### N-Back

The n-Back assesses working memory, encompassing short-term memory storage and information processing (26), and reflects “central executive” processes (27). For this visuospatial test, the 2-Back and 3-Back were used (as 1-Back is thought to assess vigilance only). The participant was asked to compare the position of a letter displayed on the screen to the position of the letter presented 2 or 3 trials previously. For example, for 2-back, the position of the 3rd letter is compared to the position of the 1 st letter and the position of the 4th letter to the 2nd letter, and so on. If the position of the letters matched, the participant pressed “M” on the keyboard for “Match” as quickly as possible. If the position of the letters did not match, the participant pressed “N” for “No Match” as quickly as possible. The first of 50 trials was presented after 1.5 s with subsequent stimulus intervals of 4.5 s. Each n-Back task was 4 mins in duration and the percentage of total accuracy was calculated. 

#### Psychomotor Vigilance Task (PVT)

The PVT is a 10 min reaction-time task of sustained attention sensitive to sleep loss (24) and can identify attentional lapses even in mild OSA (28). The device is a hand-held box with a red light-emitting diode (LED) display of a three-digit millisecond counter (PVT-192, Ambulatory Monitoring, Inc., Ardsley, NY, USA) (29). Participants were instructed to respond as fast as possible when they first saw a visual stimulus appear. The time taken to respond to the stimulus was displayed in milliseconds (ms). During the task visual stimuli appeared at random intervals between 2 to 10 s. Variables analysed were: (a) mean reaction time (RT); (b) mean of the fastest 10% of RTs; (c) mean reciprocal of slowest 10% of RTs; and, (d) number of lapses (response time >500 ms).

### Statistical Analysis

Regression using general linear models was used to evaluate differences in neurobehavioural performance outcomes between OSA and non-OSA groups while controlling for age, gender and education. All analyses were two-tailed and used an alpha level of 0.05. Mean and SD for the findings are presented as “mean (SD)”, unless otherwise noted. Variables deemed not normally distributed were transformed using appropriate methods as determined by the Box-Cox method using R. Correlations between subjective sleepiness, PSG measures of disease severity and performance were examined using Spearman’s non-parametric correlation coefficient. Data was analysed using SAS software v9.3 (SAS Institute Inc., Cary, NC, USA).

## RESULTS

Participant characteristics for OSA (*n* = 204) and non-OSA (*n* = 50) groups are shown in Table 1. During diagnostic polysomnography, the OSA group had on average a sleep efficiency of 76.9% (13.6), an apnea hypopnea index (AHI, number of apneas and hypopneas per hour of sleep) of 33.6/hr (25.8), EEG arousal index of 33.6/hr (22.3), and a 3% oxygen desaturation index (ODI) of 34.9/hr (48.0) and a minimum oxygen saturation of 78.6% (11.7). They reported on average significant levels of daytime sleepiness as measured by the ESS.

**Table 1:** Demographic and clinical features of non-OSA and OSA Groups.

	**Non-OSA *n* = 50**	**OSA *n* = 204**
Gender, n (%) male	22 (44.0%)	145 (71.1%)
Age, years [range]	39.2 (14.0) [25 – 69]	49.3 (12.5) [22 – 80]
Body Mass Index, kg/m^2^	25.7 (4.3)	33.6 (8.0)
Epworth sleepiness scale	3.6 (2.3)	12.0 (4.9)
Apnea hypopnea index, events/hour	-	33.6 (25.8)
Education Primary, n (%)	0 (0%)	5 (2.5%)
Education Secondary, n (%)	3 (6%)	54 (26.5%)
Education Tertiary, n (%)	47 (94%)	104 (51.0%)
Education Unknown, n (%)	0 (0%)	41 (20.1%)

Data are mean (SD) unless otherwise stated.

The non-OSA group had a higher proportion of females, were younger, and as expected they had a lower body mass index (BMI) and lower ESS score than the OSA group, see Table 1. A greater proportion (94%) had completed tertiary education compared with the OSA group (51%). The non-OSA group were considered low risk for the presence of sleep apnea with an average MAP index of 0.19 (0.16), and did not report clinical insomnia with an average ISI score of 3.2 (2.8).

### Neurobehavioural Performance – Comparing OSA and Non-OSA Groups

Neurobehavioural performance measures for both OSA and non-OSA groups are reported in Table 2. One hundred and sixty three patients of the 204 in the OSA group who had a known education status and all 50 non-OSA participants were included in the between–group comparisons.

**Table 2:** Comparison of neurobehavioural performance in visual spatial scanning and selective attention, executive functions and sustained attention between OSA and non-OSA groups.

**Performance Measure**	**Non-OSA *n* = 50**	**OSA *n* = 163**	**Adjusted p-value**
**Letter Cancellation Test (Visual spatial scanning and selective attention, LCT)**			
LCT Average Hits, n _(Higher Better)_	61.74 (12.39)	50.26 (13.37)	0.04
LCT Average Omissions, n _(Lower Better)_	5.27 (7.32)	3.69 (5.27)	0.02
LCT Average Commissions, n _(Lower Better)_	1.50 (2.71)	1.05 (1.77)	0.11
LCT Hits Final Trial, n _(Higher Better)_	287.58 (35.06)	281.74 (53.65)	0.64
LCT Omissions Final Trial, n _(Lower Better)_	22.42 (35.06)	28.26 (53.65)	0.64
LCT Commissions Final Trial, n _(Lower Better)_	1.08 (2.46)	4.18 (10.17)	0.37
LCT Duration Final Trial*, sec _(Lower Better)_	287.60 (70.04)	371.08 (171.38)	0.06
**Stroop Test (Inhibition of dominant responses)**			
Stroop-Text Accuracy*, % _(Higher Better)_	98.73 (2.61)	93.29 (16.51)	0.02
Stroop-Text Reaction Time, sec _(Lower Better)_	1.05 (0.29)	1.28 (0.48)	0.20
Stroop-Colour Accuracy, % _(Higher Better)_	94.43 (14.72)	79.99 (25.67)	0.08
Stroop-Colour Reaction Time, sec _(Lower Better)_	1.10 (0.60)	1.44 (0.58)	0.34
**N-Back (Working memory)**			
2-Back Accuracy*, % _(Higher Better)_	87.24 (12.60)	64.55 (26.42)	<.0001
2-Back Correct Responses, n _(Higher Better)_	42.22 (9.58)	31.06 (13.51)	<.0001
2-Back Incorrect Responses, n _(Lower Better)_	6.08 (6.49)	8.18 (7.73)	0.33
2-Back Missed Responses, n _(Lower Better)_	3.77 (8.48)	11.29 (14.79)	0.02
3-Back Accuracy, % _(Higher Better)_	80.57 (14.22)	55.69 (24.29)	<.0001
3-Back Correct Responses, n _(Higher Better)_	38.06 (11.14)	26.69 (12.30)	<.0001
3-Back Incorrect Responses, n_(Lower Better)_	8.71 (6.73)	11.04 (7.81)	0.17
3-Back Missed Responses, n _(Lower Better)_	4.29 (10.24)	13.26 (17.80)	0.02
**Psychomotor Vigilance Task (Sustained attention, PVT)**			
PVT Mean RT*, msec _(Lower Better)_	272.57 (61.92)	303.01 (76.42)	0.0003
PVT Mean Fastest 10% RT, msec _(Lower Better)_	195.00 (23.10)	204.30 (25.93)	0.06
PVT Mean Slowest 10% RRT, rate per 1000 msec _(Higher Better)_	2.57 (0.51)	2.23 (0.64)	<.0001
PVT Lapses*, n _(Lower Better)_	1.64 (3.12)	4.19 (6.09)	<.0001

P value determined by linear regression adjusted for age, gender and education. *denotes outcomes that were transformed using a Box-Cox transformation. RT, reaction time; RRT, reciprocal reaction time. For non-OSA group: *n* = 48 for LCT, *n* = 50 for Stroop, *n* = 49 for n-back, and *n* = 50 for PVT. For OSA group: *n* = 159 for LCT, *n* = 159 for Stroop, *n* = 160 for n-back, and *n* = 142 for PVT.

After controlling for age, gender and education, the OSA group demonstrated significantly impaired task performance compared with the non-OSA group in executive functions (Stroop-Text), working memory (n-Back) and sustained attention (PVT), see Table 2. In the Letter Cancellation Test the OSA patients had worse performance with fewer average hits compared with the non-OSA group. However they showed better accuracy with fewer missed targets.

### Correlations Between Subjective Sleepiness, Disease Severity and Neurobehavioural Performance

In a secondary analysis, we assessed associations between subjective sleepiness (ESS), and PSG-derived sleep efficiency (%) and disease severity (AHI, EEG arousal index, 3% ODI, min SaO_2_) measures, and neurobehavioural performance in all 204 patients in the OSA group, see Supplementary Table S1 for the full correlation matrix.

#### Subjective Sleepiness

Higher ESS was associated with slower reactions times on the PVT sustained attention task (mean reaction time [rho = 0.170, *p* = 0.022], mean reciprocal of slowest 10% of reaction times [rho = −0.166, *p* = 0.026] and lapses [rho = 0.168, *p* = 0.023]), but not any other task.

#### Sleep Efficiency

Reduced sleep efficiency (%) was significantly associated with fewer correctly marked targets and slower completion time for the final trial on the LCT (average hits [rho = 0.203, *p* = 0.010], final trial duration [rho = −0.161, *p* = 0.043]); slower reaction times on the Stroop-Colour test [rho = −0.158, *p* = 0.047]; worse working memory (accuracy and number of correct responses on 2 and 3 back tasks [rho = 0.176 to 0.196, *p* = 0.013 to 0.026]).

#### Disease Severity

The AHI or the EEG arousal index was not significantly correlated with any performance measure (*p* > 0.05) see Supplementary Table S1.

Worse hypoxemia measures were significantly related to poorer performance on tasks of executive function. Those who had the lowest minimum oxygen saturation levels showed lower accuracy on the Stroop-Colour task (rho = 0.169, *p* = 0.046), while those with a higher ODI had slower reaction times on the Stroop-Text (rho = 0.172, *p* = 0.038) and Stroop-Colour (rho = 0.179, *p* = 0.031) tasks.

## Discussion

Our brief computerised neurobehavioural test battery identified significant deficits in working memory, selective attention and sustained attention in patients with untreated OSA compared with the non-OSA group. This pattern of impairment parallels previous investigations of neurobehavioural performance in OSA (8, 30–32). Commonly used PSG-derived measures of sleep disordered breathing/hypoxemia or sleep fragmentation were inconsistently or weakly related to performance measures.

We deliberately selected tasks for the test battery based on sensitivity to evaluate cognitive domains negatively affected by OSA, with attention/vigilance and executive functions showing the most consistent impact (8, 30). The simple 10 min PVT (attention/vigilance) was sensitive to the effects of OSA with statistically significant between-group differences in 3 of the 4 PVT outcomes assessed. PVT impairment was associated with greater subjective sleepiness (higher ESS) in the current study, consistent with prior findings (33).

In the Stroop test, performance was significantly impaired on the Stroop-Text but not the Stroop-Colour component in the OSA patients. This may reflect impairment in attentional capacity and processing speed rather than an inhibition of stereotypical responses. However there was a trend for lower accuracy in Stroop-Colour in the OSA group (*p* = 0.08). Naegele et al. reported significant abnormalities in Stroop-Colour suggesting frontal lobe deficits in a group of severe OSA patients compared with age-matched controls (34). This discrepancy likely reflects differences in OSA disease severity between the populations studied, and the heterogeneity of performance amongst patients. Both sleep disruption and blood gas abnormalities have been implicated in the dysfunction of the pre-frontal cortex manifesting as “executive dysfunctions” (2). Underlying levels of alertness and attention, considered to be “lower-level cognitive processes” also influence executive functioning and response to treatment (35). Excessive daytime sleepiness is often an important factor underlying attentional deficits and neurobehavioural dysfunction (16, 27, 36), and a significant contributor to the 2-fold increased risk of workplace (4) and motor vehicle accidents (5) in those with OSA. Importantly, the largest improvements in cognitive deficits following CPAP treatment are observed in patients who are excessively sleepy at baseline (12).

To delineate the role of sleep disruption and hypoxemia on neurobehavioural dysfunction we examined the association between nocturnal PSG measures and performance in 204 OSA patients. We found inconsistent findings and weak associations, similar to those reported by others (9, 37–39). Reduced sleep efficiency was significantly but weakly associated with poor performance on the selective attention (LCT), executive function (Stroop-Text and Stroop-Colour) and working memory tasks. A lower minimum oxygen saturation level and ODI were associated with impaired executive functioning with reduced accuracy and slower reaction times on the Stroop task, respectively. The AHI and the EEG arousal index were not significantly related to any performance measure. The length of time individuals have been affected by OSA is often unclear and is a likely factor in the magnitude and reversibility of performance deficits, and may explain, at least in part, the inconsistencies with performance and disease severity.

In the selective attention task (LCT), OSA patients had fewer average hits but were more accurate compared with the non-OSA group. There was also a trend for the OSA patients to take longer to complete the final combined trial (*p* = 0.06), possibly reflecting less impulsivity and “appreciation of the complexity of the task” (2) often associated with increasing age. However it may also highlight the wide variation in deficits and performance more resilient to the effects of untreated OSA. Previous research found increasing age in healthy adults (age range of 18–91 years) resulted in similar LCT deficits in speed but not in spatial distribution of cancellation errors (40). Although group differences could be explained by differences in age (41) and education, we controlled for these variables, suggesting these deficits are more likely to relate to the presence of untreated OSA.

Prior neuroimaging research has provided insight into the OSA-related deficits observed in higher-level cognitive functions such as those targeted by the current battery, and the mechanisms which underlie them. Using functional MRI, altered brain responses have been identified during the completion of response inhibition and working memory tasks in OSA (42–45), including reduced activation in the prefrontal cortex with working memory (45). When OSA patients performed equally well to controls on 2-back, an over-recruitment of brain regions during the task was apparent with increased activation in the frontal cortex and hippocampus (44). This lack of activation of the prefrontal cortex potentially indicates injury to this area with a compensatory over-recruitment of the frontal cortex and hippocampus. Interestingly some OSA patients appear able to reallocate or recruit additional neuronal resources to maintain comparable executive functioning performance to controls (44, 46).

There are a number of study limitations including using a previously-collected data set constructed for a CPAP adherence research outcome. However, the parsimonious use of the 204 prior patients and 50 non-OSA participants is an important ethical use of the data, and is sufficiently large to detect clinically meaningful differences between the OSA patients and non-OSA participants. Our non-OSA healthy comparison group was less sleepy, had a lower BMI, was younger, and had a higher percentage of females and with more tertiary education than in the OSA group. It is difficult to control for potential differences in BMI and sleepiness as these are both contributing factors to the disease in untreated OSA. As this clinical OSA cohort are more likely to have presented to the sleep centre for management of symptoms of OSA, including neurobehavioural impairment some selection bias is possible. However these individuals were primarily recruited to explore the effects of a psychoeducation program to improve CPAP adherence and any self-selection bias to determine any cognitive deficits is likely to be minimal. While the non-OSA group was not individually-matched to the OSA group for gender, age and education, the differences between groups persisted after statistical modelling adjusting for these potential confounders. The non-OSA group did not undergo polysomnography to rule out the presence of sleep apnea, however the screening tools of the MAP index and ESS showed data consistent with that reported from normal controls without OSA (20, 21). There was no single neurobehavioural performance outcome nominated as the pre-specified primary outcome. We specifically chose to evaluate all component outcomes for each task to determine the sensitivity of the test battery to detect differences between OSA and non-OSA groups. As a consequence, multiple comparisons were conducted increasing the likelihood of falsely detecting a between-group difference (type I error). Lastly, we did not assess more complex components of executive functioning such as problem solving and planning in order to maintain the brevity of the test battery.

The currently developed battery is well placed to examine the negative effects of OSA and effectively detect between-group differences in cognitive performance. It has potential, not only in terms of its ease of application without loss of fidelity, but also in the long term, to broaden our knowledge of the variability in the manifestations of the disease between patients, and assess response to treatment. This brief 30 min assessment may provide a sensitive, standardised method of assessing daytime dysfunction in OSA. However, to date it has not yet been subjected to more rigorous psychometric evaluation, and further research is now required to document the reliability and validity of these tools in OSA.

## Ethics Statement

This work was carried out in accordance with the recommendations of the ICH-GCP guidelines and the Australian National Statement on Ethical Conduct in Human Research with written informed consent from all subjects. All subjects gave written informed consent in accordance with the Declaration of Helsinki. The study protocols were approved by the Human Ethics Review Committees of the Sydney South West Area Health Service and the University of Sydney.

## Author Contributions

Study design: RG, DB, KW, AD. Data collection: DJB, ALD. Data analysis: CF, CH, KW, GD. Interpretation of results: AD, DB, KW, CF, CH, SN, GD. Drafting of the manuscript: AD, CF, DB, CH, KW, RG, SN and all other authors contributed to the final manuscript.

## Conflict of Interest Statement

The authors declare that the research was conducted in the absence of any commercial or financial relationships that could be construed as a potential conflict of interest.

## Supplementary material

Table S1 Correlations between subjective sleepiness, disease severity and neurobehavioural performance.
