# Supplementary material for: Impaired Neurobehavioural Performance in Untreated Obstructive Sleep Apnea Patients Using a Novel Standardised Test Battery
Source: Front Surg. 2018 May 18;5:35. doi: 10.3389/fsurg.2018.00035 (PMC5968192; doi:10.3389/fsurg.2018.00035)
Supplement: Table S1 — Correlations between subjective sleepiness, disease severity and neurobehavioural performance. [file Table1.DOCX]

# Supplementary Table 1: Correlations between subjective sleepiness, disease severity and neurobehavioural performance

|  |  | **Epworth sleepiness scale** | **Sleep efficiency** | **Apnea hypopnea index** | **EEG arousal index** | **3% oxygen desaturation index** | **Minimum oxygen saturation** |
| --- | --- | --- | --- | --- | --- | --- | --- |
| **LCT Average Hits** | **rho** | 0.026 | 0.203* | -0.073 | -0.018 | -0.109 | 0.064 |
|  | ***p-value*** | *0.721* | *0.010* | *0.380* | *0.828* | *0.191* | *0.451* |
| **LCT Average Omissions** | **rho** | -0.005 | -0.039 | 0.032 | -0.001 | -0.071 | 0.023 |
|  | ***p-value*** | *0.941* | *0.629* | *0.699* | *0.994* | *0.397* | *0.785* |
| **LCT Average Commissions** | **rho** | 0.068 | 0.008 | 0.043 | 0.040 | 0.087 | -0.050 |
|  | ***p-value*** | *0.344* | *0.919* | *0.606* | *0.619* | *0.298* | *0.561* |
| **LCT Hits Final Trial** | **rho** | -0.005 | -0.036 | -0.056 | -0.046 | -0.027 | 0.041 |
|  | ***p-value*** | *0.945* | *0.654* | *0.499* | *0.572* | *0.751* | *0.630* |
| **LCT Omissions Final Trial** | **rho** | -0.017 | 0.070 | 0.027 | 0.019 | -0.001 | -0.003 |
|  | ***p-value*** | *0.810* | *0.378* | *0.744* | *0.817* | *0.994* | *0.977* |
| **LCT Commissions Final Trial** | **rho** | 0.005 | 0.008 | 0.086 | 0.006 | 0.096 | -0.161 |
|  | ***p-value*** | *0.946* | *0.917* | *0.300* | *0.939* | *0.249* | *0.057* |
| **LCT Duration Final Trial** | **rho** | 0.023 | -0.161* | -0.007 | -0.059 | 0.059 | -0.154 |
|  | ***p-value*** | *0.745* | *0.043* | *0.938* | *0.465* | *0.484* | *0.069* |
| **Stroop-Text Accuracy** | **rho** | -0.105 | -0.041 | -0.117 | -0.041 | -0.031 | 0.092 |
|  | ***p-value*** | *0.141* | *0.607* | *0.157* | *0.610* | *0.710* | *0.276* |
| **Stroop-Text Reaction Time** | **rho** | -0.054 | -0.086 | 0.093 | 0.013 | 0.172* | -0.102 |
|  | ***p-value*** | *0.452* | *0.280* | *0.259* | *0.868* | *0.038* | *0.228* |
| **Stroop-Colour Accuracy** | **rho** | 0.004 | 0.134 | -0.073 | -0.066 | -0.074 | 0.169* |
|  | ***p-value*** | *0.961* | *0.091* | *0.376* | *0.411* | *0.375* | *0.046* |
| **Stroop-Colour Reaction Time** | **rho** | -0.096 | -0.158* | 0.042 | 0.010 | 0.179* | -0.102 |
|  | ***p-value*** | *0.180* | *0.047* | *0.608* | *0.902* | *0.031* | *0.228* |
| **2-Back Accuracy** | **rho** | -0.027 | 0.176* | -0.100 | -0.072 | -0.005 | 0.070 |
|  | ***p-value*** | *0.705* | *0.026* | *0.223* | *0.367* | *0.956* | *0.405* |
| **2-Back Correct Responses** | **rho** | -0.020 | 0.179* | -0.083 | -0.061 | 0.012 | 0.027 |
|  | ***p-value*** | *0.779* | *0.024* | *0.316* | *0.449* | *0.888* | *0.753* |
| **2-Back Incorrect Responses** | **rho** | 0.061 | -0.116 | 0.001 | 0.054 | 0.026 | 0.004 |
|  | ***p-value*** | *0.393* | *0.145* | *0.995* | *0.506* | *0.759* | *0.964* |
| **2-Back Missed Responses** | **rho** | 0.024 | -0.123 | 0.130 | 0.068 | 0.025 | -0.115 |
|  | ***p-value*** | *0.735* | *0.121* | *0.114* | *0.397* | *0.765* | *0.173* |
| **3-Back Accuracy** | **rho** | -0.030 | 0.196* | -0.082 | -0.009 | -0.051 | 0.030 |
|  | ***p-value*** | *0.672* | *0.013* | *0.320* | *0.911* | *0.538* | *0.724* |
| **3-Back Correct Responses** | **rho** | -0.001 | 0.192* | -0.059 | 0.004 | -0.065 | 0.022 |
|  | ***p-value*** | *0.991* | *0.015* | *0.477* | *0.963* | *0.434* | *0.793* |
| **3-Back Incorrect Responses** | **rho** | 0.108 | -0.117 | -0.036 | 0.026 | -0.006 | 0.041 |
|  | ***p-value*** | *0.131* | *0.141* | *0.665* | *0.745* | *0.947* | *0.630* |
| **3-Back Missed Responses** | **rho** | -0.001 | -0.117 | 0.106 | 0.017 | 0.110 | -0.113 |
|  | ***p-value*** | *0.993* | *0.141* | *0.197* | *0.831* | *0.188* | *0.179* |
| **PVT Mean RT** | **rho** | 0.170* | 0.030 | -0.026 | -0.122 | 0.099 | 0.009 |
|  | ***p-value*** | *0.022* | *0.719* | *0.762* | *0.141* | *0.250* | *0.921* |
| **PVT Mean Fastest 10% RT** | **rho** | 0.057 | -0.074 | -0.077 | -0.146 | 0.035 | 0.076 |
|  | ***p-value*** | *0.447* | *0.365* | *0.364* | *0.077* | *0.685* | *0.389* |
| **PVT Mean Slowest 10% Reciprocal RT** | **rho** | -0.166* | -0.060 | 0.018 | 0.108 | -0.076 | -0.003 |
|  | ***p-value*** | *0.026* | *0.462* | *0.830* | *0.193* | *0.375* | *0.972* |
| **PVT Lapses** | **rho** | 0.168* | 0.050 | 0.013 | -0.142 | 0.074 | -0.030 |
|  | ***p-value*** | *0.023* | *0.537* | *0.878* | *0.085* | *0.390* | *0.732* |
| EEG, electroencephalogram; LCT, letter cancellation test; PVT, psychomotor vigilance task; RT, reaction time. Correlations are Spearman’s rho. * p < 0.05 | | | | | |  |  |
